# Deciphering the role of epigenetic modifications in fatty liver disease: A systematic review

**DOI:** 10.1111/eci.13479

**Published:** 2021-01-04

**Authors:** Xiaofang Zhang, Eralda Asllanaj, Masoud Amiri, Eliana Portilla‐Fernandez, Wichor M. Bramer, Jana Nano, Trudy Voortman, Qiuwei Pan, Mohsen Ghanbari

**Affiliations:** ^1^ Department of Epidemiology Erasmus MC Erasmus University Medical Center Rotterdam the Netherlands; ^2^ Institute for Community Medicine University Medicine Greifswald Greifswald Germany; ^3^ Medical Library Erasmus MC Erasmus University Medical Centre Rotterdam the Netherlands; ^4^ Institute of Epidemiology Helmholtz Zentrum München German Research Center for Environmental Health Neuherberg Germany; ^5^ German Diabetes Center München‐Neuherberg Germany; ^6^ Department of Gastroenterology and Hepatology Erasmus MC Erasmus University Medical Center Rotterdam the Netherlands

**Keywords:** DNA methylation, epigenetics, microRNAs, NAFLD, nonalcoholic steatohepatitis

## Abstract

**Background:**

Fatty liver disease (FLD), primarily nonalcoholic fatty liver disease (NAFLD), is the most common liver disorder that affects a quarter of the global population. NAFLD is a spectrum of disease ranging from simple steatosis to nonalcoholic steatohepatitis, which is associated with increased risk of developing liver cancer. Given that the pathogenic mechanisms of fatty liver remain largely elusive, it is important to further investigate potential underlying mechanisms including epigenetic modifications. Here, we performed a systematic review of human epigenetic studies on FLD presence.

**Methods:**

Five bibliographic databases were screened until 28 August 2020. We included cross‐sectional, case‐control and cohort studies in humans that examined the association of epigenetic modifications including global, candidate or epigenome‐wide methylation of DNA, noncoding RNAs and histone modifications with FLD.

**Results:**

In total 36 articles, based on 33 unique studies, consisting of 12 112 participants met the inclusion criteria. Among these, two recent epigenome‐wide association studies conducted among large population‐based cohorts have reported the association between cg06690548 (*SLC7A11*) and FLD. Moreover, several studies have demonstrated the association between microRNAs (miRNAs) and FLD, in which miR‐122, miR‐34a and miR‐192 were recognized as the most relevant miRNAs as biomarkers for FLD. We did not find any studies examining histone modifications in relation to FLD.

**Conclusions:**

Cumulative evidence suggests a link between epigenetic mechanisms, specifically DNA methylation and miRNAs, and FLD. Further efforts should investigate the molecular pathways by which these epigenetic markers may regulate FLD and also the potential role of histone modifications in FLD.

## 1 INTRODUCTION

Fatty liver disease (FLD), also called hepatic steatosis, is defined as intrahepatic fat of at least 5% of liver weight. The majority of fatty liver patients develop nonalcoholic fatty liver disease (NAFLD), which is the most common cause of chronic liver disease worldwide. Currently, the prevalence is about 25% of the global population with the highest burden among Middle Eastern and South American countries.^1^ NAFLD is a spectrum of disease ranging from simple steatosis, which has a negligible risk of progression to cirrhosis, to nonalcoholic steatohepatitis (NASH), which has an increased risk of progression to cirrhosis and eventually liver cancer.^2^ The molecular mechanisms underlying these processes are not entirely understood. Further investigations that could provide a better understanding of the disease pathogenic mechanisms are important to improve early diagnosis and treatment of FLD.

The pathogenesis of FLD is multifactorial. Exposure to particular environmental factors lifestyle habits, nutritional factors and genetics are thought to influence the disease risk, progression and prognosis. Emerging evidence suggests that epigenetic modifications may also contribute to the pathophysiology of FLD.^3^ Epigenetics including DNA methylation, histone modifications and noncoding RNAs refers to stable and heritable alterations in regulating gene expression, independent of changes in the DNA sequence.^4^ Among noncoding RNAs, microRNAs (miRNAs), the small noncoding RNA molecules that regulate gene expression at the post‐transcriptional level, are the most extensively studied epigenetic markers in regard to FLD risk.^5^ Many studies have explored the role of miRNAs in the pathogenesis of FLD and their potential as biomarkers of the disease, but the results are sometimes inconsistent.^6^, ^7^, ^8^, ^9^ Long noncoding RNAs (lncRNAs) are a group of RNA molecules longer than 200 bases without protein‐coding capacity, involved in chromatin remodelling, as well as transcriptional and post‐transcriptional gene regulation.^10^ They have been mainly studied in mouse models of NAFLD or NASH with a few studies conducted in human. DNA methylation is another important epigenetic mechanism that has been suggested to contribute to the pathophysiology of fatty liver disease.^4^ The three most common approaches to investigate the association of DNA methylation signatures with a trait of interest are global DNA methylation, candidate gene approach and epigenome‐wide association studies (EWAS). Unlike genetic variation, epigenetic modifications comprise dynamic changes and potentially reversible; therefore, it could be modified by lifestyle and other therapeutic approaches.

Previous studies have summarized the evidence pertaining epigenetic mechanisms and FLD.^5^, ^11^ However, these studies mainly focused on individual epigenetic mechanism, and therefore a comprehensive assessment of other epigenetic modifications such as DNA methylation, histone modifications present in FLD is currently lacking. Thus, this study aimed to conduct a systematic review of the current evidence in human studies to comprehensively evaluate the association between epigenetic modifications and FLD.

## 2 METHODS

### 2.1 Data sources and search strategies

This systematic review was conducted using a predesigned protocol and was reported in accordance with PRISMA^12^ guidelines (Table S1). The studies published until 28 August 2020 (date last searched) were searched in five bibliographic databases: Embase.com, Medline ALL (Ovid), Web of Science Core Collection, Cochrane Central Register of trials and Google scholar. The search was performed by an experienced medical information specialist (WMB). In Embase.com and Medline (Ovid) databases, articles were searched by thesaurus terms, title and/or abstract; in other databases, only by title and/or abstract. The search combined terms related to the exposure (eg epigenetics, DNA methylation, histone modifications, noncoding RNAs and microRNAs) and outcome (eg fatty liver, NAFLD, alcoholic liver disease, nonalcoholic and NASH). The search was restricted only to studies conducted on humans. The full search strategy is provided in Table S2.

### 2.2 Study selection and inclusion criteria

Studies were eligible for inclusion if they (a) were cross‐sectional, case‐control or cohort studies; (b) assessed epigenetic marks (global, candidate gene studies or epigenome‐wide analysis methylation of DNA, noncoding RNAs, miRNAs or histone modifications); (c) were conducted in humans; (d) collected data on FLD (fatty liver disease, NAFLD, hepatic steatosis, hepatic fat, simple steatosis and NASH) and (e) reported the association of any of the above‐mentioned epigenetic marks with FLD.

We screened the retrieved titles and abstracts and selected eligible studies according to the predefined selection criteria (Table S3). The full texts of the selected records which satisfied selection criteria were obtained and examined further by two researchers (XZ and EA). In case of disagreement, decision was made through consensus or consultation with a third independent reviewer (MA). Full texts were retrieved for studies that satisfied all selection criteria.

### 2.3 Data extraction and quality assessment

Data extraction and quality assessment were independently conducted by two researchers (XZ and EA) using a predesigned form. The form included information on study authors, publication date, population groups with mean age, sample sizes, geographical location, study design, outcome, tissue type, adjustments/ matching, main findings and quality of study. Potential bias within each individual study was evaluated by two independent reviewers (XZ and EA) using the validated Newcastle‐Ottawa Scale (NOS),^13^ a semi‐quantitative scale designed to evaluate the quality of case‐control or cohort studies. We evaluated cross‐sectional studies using an adapted version of the scales. Study quality was judged based on these items: the selection criteria of participants, comparability of cases and controls, and exposure and outcome assessments. The NOS assigns a maximum of 4 points for selection, 2 points for comparability and 3 points for exposure or outcome, with 9 points referring to highest quality of the study and to be at low risk of bias. Studies scoring 1‐3 were defined as low, 4‐6 as average and 7‐9 as high quality.

## 3 RESULTS

**FIGURE 1:**
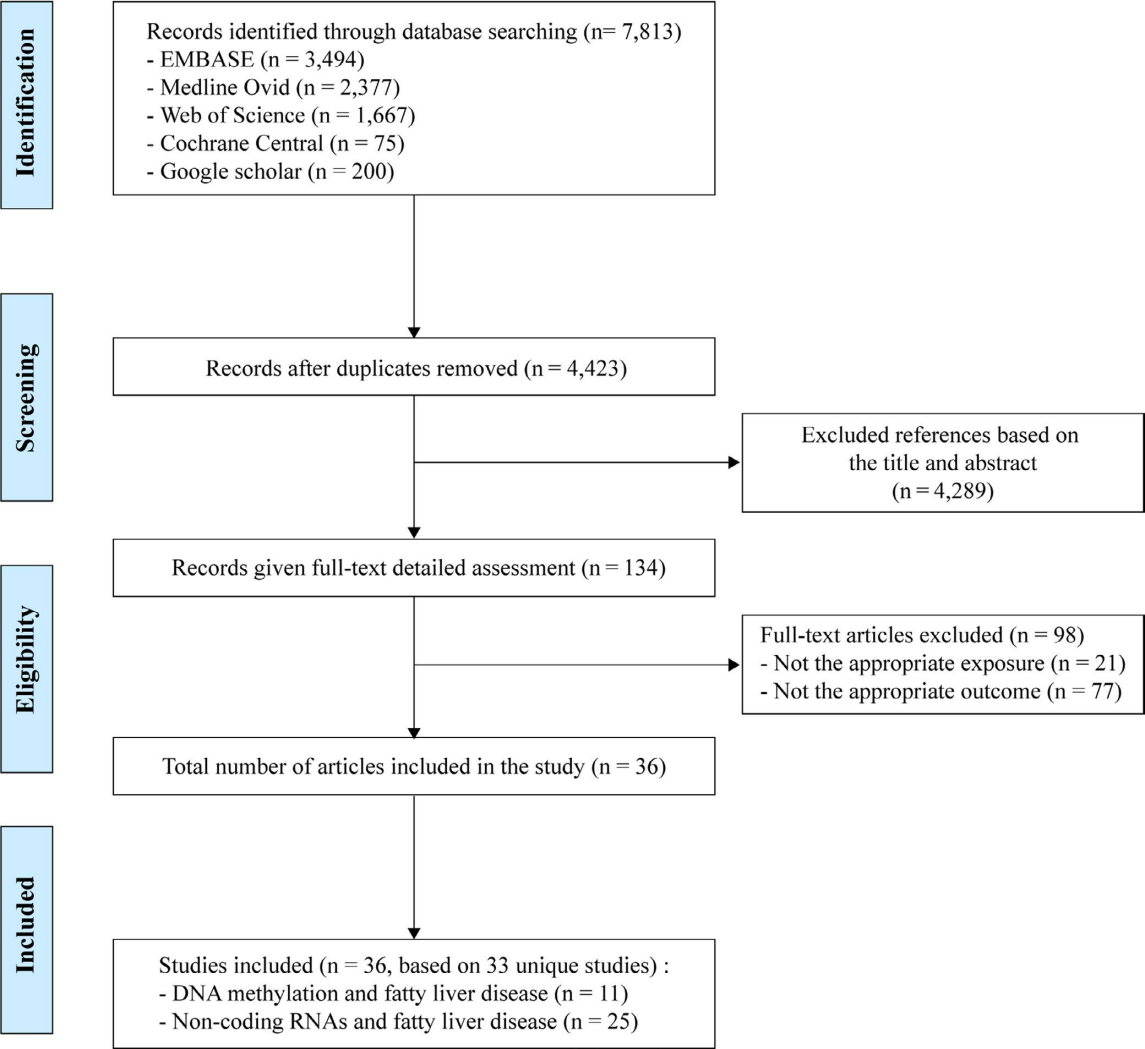
Flow chart of studies included in the systematic review

As shown in Figure 1, 7813 potentially relevant records were identified from five databases. After removing duplicates, 4423 records were retained. Of these, 4289 records were excluded based on titles and abstracts. For the remaining 134 records, full‐text articles were reviewed, 98 of which were excluded for various reasons as described in Figure 1. A total of 36 articles met the eligibility criteria and were included in this review. In the following section, a summary of all the included studies is provided, followed by a review of their findings. Results are presented for DNA methylation (including the global, candidate gene analysis and EWAS approach) and noncoding RNAs (miRNAs and lncRNAs).

### 3.1 Summary of included studies

Of the 36 included publications (33 unique studies), two studies assessed global DNA methylation, five studies assessed DNA methylation for specific candidate genes and four studies used the EWAS approach. Moreover, 24 studies investigated miRNAs and one studied lncRNAs. There were no studies examining histone modifications in relation to FLD.

A total of 12 112 individuals were involved in all the studies. The mean age across all studies was 48.8 years and included participants from Asian (n = 13), European (n = 11), North American (n = 7), South American (n = 4) ancestries and one study included subjects from both European and North American ancestries.^14^ Overall, the study designs were as follows: cohort (n = 16), case‐control (n = 10), cross‐sectional (n = 9) and one study including both a cross‐sectional and a prospective cohort design. Epigenetic signatures were measured in liver tissue (n = 10), blood samples (n = 16) and both liver and blood (n = 10). The studies included in this review diagnosed FLD based on two different methods: measured by liver biopsy (n = 19) or by imaging (including ultrasonography, liver magnetic resonance imaging and computed tomography) (n = 17). The majority of articles focused on miRNAs and NAFLD mainly used qPCR‐based methods to measure the expression levels of miRNAs, while fewer used next‐generation sequencing. Most of the candidate gene DNA methylation studies used bisulphite pyrosequencing, a quantitative approach with high reproducibility, but with relatively short length of reads.^15^ The most commonly platform used in the EWAS publications was the Illumina Infinium Human Methylation 450 Bead Chip, which enabled the screening of over 450 000 CpGs with high quantitative accuracy. Detailed characteristics of the 36 included studies are summarized in Tables 1 and 2.

**TABLE 1:** Overview of included studies on DNA methylation and fatty liver disease

Lead Author, Year of publication	Mean age, Sample size, Country	Study design	Outcome	Measurement method	Tissue type	Adjustment level/Matching	Main findings	Study quality^a^
Global DNA methylation and fatty liver disease								
Pirola et al, 2013^16^	49.4, n = 63, Argentina	Case‐control	NAFLD and NASH	Methylation‐specific PCR and liver biopsy	Liver tissue	None	MT‐ND6 methylation was higher in the liver of NASH than simple steatosis patients (*P* < .04) and the methylation level of MT‐ND6was significantly associated with NAFLD activity score (*P* < .02).	6
Mello et al, 2017^17^	49.5, n = 95, Finland	Cross‐sectional	NAFLD and NASH	LINE‐1 DNA methylation, Bisulphite pyrosequencing and liver biopsy	Liver tissue	BMI, age, sex and T2D	NASH was associated with LINE‐1 hypomethylation compared with simple steatosis or normal liver.	8

Lead Author, Year of publication	Mean age, Sample size, Country	Study design	Outcome	Methylation sites, Measurement method	Tissue type	Adjustment level/ Matching	Main findings	Study quality^a^
Candidate gene approach and fatty liver disease								
Sookoian et al, 2010^18^	50.2, n = 74, Argentina	Case‐control	NAFLD	Methylation‐specific PCR and liver biopsy	Blood and liver tissue	HOMA‐IR	The liver mtDNA/nDNA ratio was significantly higher in control livers compared with NAFLD livers. mtDNA/nDNA ratio was inversely correlated with *PPARGC1A* promoter methylation.	7
Kordi‐Tamandani et al, 2011^19^	39.2, n = 160, Iran	Case‐control	NAFLD	*GSTT1* and *GSTP1*, Methylation‐specific PCR and ultrasonography	Peripheral blood sample	None	No association between methylation status and expression profiles of *GSTT1* and *GSTP1* genes and NAFLD.	6
Murphy et al, 2013^20^	51.2, n = 90, USA	Cohort	NAFLD	*FGFR2*, *MAT1A*, and *CASP1*, Bisulphite pyrosequencing and liver biopsy	Liver tissue	None	Most of differentially methylated CpG sites were hypomethylated in obese patients with advanced vs mild NAFLD. Methylation at *FGFR2*, *MAT1A* and *CASP1* was validated in a replication cohort.	6
Kitamoto et al, 2015^21^	53.7, n = 95, Japan	Cohort	NAFLD	*PNPLA3*, *SAMM50*, and *PARVB*, Targeted‐bisulphite sequencing and liver biopsy	Liver tissue	None	*PARVB* was hypomethylated in the livers of patients with advanced NAFLD, *PNPLA3* was hypermethylated in these patients. The levels of SAMM50 mRNA did not differ between patients with mild or advanced NAFLD.	6

Lead Author, Year of publication	Mean age, Sample size, Country	Study design	Outcome	Methylation sites, Measurement method	Tissue type	Adjustments/Matching	Main findings	Study quality^a^
Zeybel et al, 2015^22^	59.9, n = 34, UK	Cross‐sectional	NAFLD	*PPARα*, *TGFβ1*, *Collagen 1A1* and *PDGFα*, Bisulphite pyrosequencing and liver biopsy	Liver tissue	None	DNA methylation at specific CpG sites within genes known to affect fibrogenesis distinguishes between patients with mild severe fibrosis in NAFLD.	6
Epigenome‐wide association studies and fatty liver disease								
Hotta et al, 2017^25^	51.1, n = 60, Japan	Cohort	NAFLD	Illumina Infinium Human Methylation 450 BeadChip and liver biopsy	Liver tissue	None	four hub genes (*PAPLN, LBH, DPYSL3, and JAG1)* overexpressed in advanced NAFLD.	6
Mwinyi et al, 2017^24^	51.0, n = 178, Sweden	Cohort	NAFLD	Illumina Human Methylation 450 Beadchip and liver biopsy	Liver tissue	Gender, age, BMI and liver disease state	NAFLD is associated with methylation shifts with relevance for the expression of *NPC1L1*, *STARD* and *GRHL* involved in lipoprotein particle composition.	8
Nano et al, 2017^23^	63.5, n = 1450, The Netherlands	Cohort	Hepatic steatosis	Illumina Human Methylation 450K array and ultrasound	Whole blood samples	Age, sex, cohort, lifestyle factors, BMI, hypertension, triglycerides, HDL, glucose level, liver enzyme levels.	DNA methylation at cg06690548 (*SLC7A11*) was associated with reduced risk of hepatic steatosis in participants (odd ratio, 0.69; 95%CI = 0.55‐0.93; *P*‐value = 2.7 × 10^−3^)	8
Ma et al, 2019^14^	61.2, n = 4525, USA and the Netherlands	Cross‐sectional and cohort	Hepatic fat and NAFLD	Illumina Infinium HumanMethylation450 (450K) BeadChip, CT and ultrasound	Whole blood samples	Age, sex, smoking status, physical activity levels, and alcohol intake and BMI	22 CpGs associated with hepatic fat in European ancestry participants. Mendelian randomization analyses supported the association of hypomethylation of cg08309687 (*LINC00649*) with NAFLD (*P* = 2.5 × 10^−4^).	8

Abbreviations: BMI, body mass index; CT, computed tomography; HDL, high‐density lipid; HOMA‐IR, homeostatic model assessment‐insulin resistance; LINE, Long‐interspersed nuclear element; mtDNA, mitochondrial DNA; MT‐ND6, mitochondrially encoded NADH dehydrogenase 6; NAFLD, nonalcoholic fatty liver disease; NASH, nonalcoholic steatohepatitis;nDNA, nuclear DNA; PCR, polymerase chain reaction; T2D, type 2 diabetes.

^a^ Quality assessment based on the Newcastle‐Ottawa Scale. Range 0‐9, higher score is higher quality.

**TABLE 2:** Overview of included studies on noncoding RNAs and fatty liver disease

Author, Year of publication	Mean age, Sample size, Country	Study design	Outcome	Measurement method	Adjustments/Matching	Main findings	Study quality^a^
miRNAs and fatty liver disease measured in blood samples							
Yamada et al, 2013^6^	66.1, n = 403, Japan	Cross‐sectional	NAFLD	qRT‐PCR and ultrasound	None	MiR‐21, miR‐34a, miR‐122, and miR‐451 were higher in NAFLD patients. MiR‐34a may present a therapeutic target for NAFLD.	6
Becker et al, 2015^26^	41.4, n = 198, Germany	Cohort	NAFLD and NASH	RT‐PCR and ultrasound	Age, gender, BMI, *CK18‐Asp396*, miR‐122, miR‐192, miR‐223, miR‐21	MiR‐122 and miR‐192 to be differentially regulated in NAFLD. MiR‐21 upregulated in participant with NASH than healthy controls.	8
Xu et al, 2015^27^	50.0, n = 80, China	Case‐control	NAFLD	miRNA microarray analyses and ultrasound	Age	MiR‐103 may be a molecular link between insulin resistance and NAFLD and a therapeutic target for these disorders.	7
Mehta et al, 2016^28^	62.5, n = 44, USA	Cohort	NAFLD	qPCR and ultrasound	None	Obese patients with NAFLD, lower circulating levels of miR‐145, miR‐211, miR‐146a and miR‐30c than lean with NAFLD. miR‐161 and miR‐241, higher levels in the obese patients with NAFLD than lean with NAFLD.	6
Zarrinpar et al, 2016^29^	48.5, n = 80, USA	Cross‐sectional	NAFLD	qRT‐PCR and liver MRI	None	MiR‐331‐3p and miR‐30c, were different between NAFLD and healthy controls (for miR‐331‐3p: 7.644 ± 0.091 vs 8.057 ± 0.071, *P* = .004; for miR‐30c: 10.013 ± 0.126 vs 10.418 ± 0.086, *P* = .008).	6
Raitoharju et al, 2016	42.4, n = 871, Finland	Cohort	NAFLD and FLD	TaqMan OpenArray miRNA panel and ultrasonography	MiR‐122‐5p or miR‐885‐5p, age, sex, BMI, TG, insulin levels, blood pressure, lifestyle factors	MiR‐122‐5p and miR‐885‐5p may be associated with fatty liver formation through the regulation of lipoprotein metabolism.	8
Abdel‐Hamed et al, 2017^30^	40.0, n = 150, Egypt	Case‐control	NAFLD	qRT‐PCR and ultrasonography	None	Serum miRNA‐122 expression showed positive association with increased susceptibility to NAFLD in the study population.	6
Brandt et al, 2018^31^	10.1, n = 147, Germany, Italy and Slovenia	Cohort	NAFLD	qPCR and Liver ultrasonography,	None	MiR‐122 levels were higher in children with NAFLD compared with healthy controls.	6
He et al, 2019^32^	57.5, n = 276, China	Cohort	NAFLD	qPCR and abdominal ultrasonography,	Age, sex, and BMI	Serum miR‐29b was positively associated with NAFLD (odds ratio 2.04 [1.16‐3.58], *P* = .013).	8
Ando et al, 2019^33^	63.8, n = 475, Japan	Cross‐sectional	NAFLD	qRT ‐PCR and ultrasonography	age, sex, BMI, SBP, HbA1c, TG, LDL‐c, eGFR, cigarette smoking status and medication history	Down‐regulated circulating miR‐20a and miR‐27a levels were significantly associated with severe NAFLD in the general population. Circulating miR‐20a and miR‐27a may be useful biomarkers for severe NAFLD.	8
Hendy et al, 2019^34^	41.5, n = 300, Egypt	Case‐control	NAFLD	RT‐qPCR and abdominal ultrasonography	Age and gender	Compared with the control subjects, both miRNA‐122 and miR‐34a levels were increased in NAFLD (*P* < .01) and at a cut‐off = 1.261, miRNA‐122 had 92% sensitivity, 85% specificity to differentiate NAFLD from healthy controls, while miRNA‐99a were significantly decreased in NAFLD	8
Delik et al, 2020^35^	46.3, n = 60, Turkey	Case‐control	NAFLD	SYBR Green based quantitative and various imaging procedure	None	No statistically significant results were found between miRNA‐122 levels and participants with NAFLD compare to control group (*P* = .090). No significant results were found between patient and control group for *PNPLA3* I148M polymorphism (*P* = .087).	6
Hu et al, 2020^36^	52.2, n = 240, China	Case‐control	NAFLD	qRT ‐PCR and ultrasonography	Age and gender	Serum expression of miR‐192‐5p in acute pancreatitis patients with NAFLD is significantly decreased and serves as a candidate diagnostic biomarker.	8
miRNAs and fatty liver disease measured in liver tissue							
Sharma et al, 2013^37^	46.0, n = 24, USA	Cohort	NAFLD and NASH	RT‐qPCR and liver biopsy	Age and sex	Both NASH and ballooning degeneration of hepatocytes correlated negatively with the expression levels of miR‐125b. Histologic NASH correlated positively with the expression levels of miR‐16‐2 and miR‐7‐1.	8
Braza‐Boïls et al, 2016^2^	41.5, n = 239, Spain	Cohort	NAFLD and NASH	qRT‐PCR and liver biopsy	Age, BMI and abdominal circumference	An increase in miR‐34a‐5p and a decrease in miR‐122‐5p and miR‐29c‐3p in patients with NASH vs controls without NAFLD were observed (*P* < .05).	8
Auguet et al, 2016^38^	46.6, n = 122, Spain	Cohort	NAFLD and NASH	RT‐qPCR and liver biopsy	Age, BMI, HDL cholesterol, triglycerides, AST and ALT	In obese women, higher miR‐33b* liver expression is associated with NASH. MiR‐122 circulating levels could be included in a panel of different biomarkers to improve accuracy in diagnosis of NASH.	8
miRNAs and fatty liver disease measured in both blood samples and liver tissue							
Estep et al, 2010^39^	46.0, n = 24, USA	Cohort	NAFLD and NASH	TaqMan Human MicroRNA arrays and IPA, liver biopsy	Age, race, gender, BMI and presence of diabetes mellitus	MiR‐132, miR‐150, miR‐433, miR‐28‐3p, miR‐511, miR‐517a and miR‐671 significant differentially expressed between NASH and NAFLD patients.	8
Celikbilek et al, 2014^9^	43.6, n = 40, Turkey	Cross‐sectional	NAFLD and NASH	RT‐qPCR, liver biopsy	Age	miR‐181d, miR‐99a, miR‐197 and miR‐146b were lower in NAFLD patients than in healthy controls. miR‐181d and miR‐99a were inversely correlated with serum GGT levels in NASH patients.	7
Pirola et al, 2015^40^	49.8, n = 158, Argentina	Case‐control	NASH and simple steatosis	ISH, RT‐PCR and liver biopsy	Age, BMI and fatty liver	miR‐122 and miR‐192 dramatic and significant fold changes were observed in participants with NASH compare to simple steatosis.	8
Muangpaisarn et al, 2017^8^	46.0, n = 73, Thailand	Cross‐sectional	NAFLD	RT‐PCR and liver biopsy	None	Serum level of miR‐34a may serve as a biomarker of liver inflammation and fibrosis in patients with NAFLD.	6
Liu et al, 2016^41^	40.5, n = 111, China	Cohort	NAFLD and NASH	qRT‐PCR and ultrasonography	BMI, miR‐34a	Circulating miR‐122, miR‐16, miR‐192 and miR‐34a showed differential expression levels between NAFLD and miR‐34a had an approximately 2‐fold increase in NAFLD samples compared with that of CHB samples (*P* < .01). only serum miR‐16 levels were associated with fibrosis (R = 0.350, *P* < .05) in patients with NAFLD.	8
Salvoza et al, 2016^7^	41.3, n = 64, USA	Cross‐sectional	NAFLD	qRT‐PCR and liver biopsy	None	MiR‐34a and miR‐122 are potential markers for discriminating NAFLD patients from healthy controls with an area AUC values of 0.781 and 0.858, respectively.	6
Akuta et al, 2020^42^	52 n = 441, Japan	Cohort	NAFLD	RT‐qPCR and liver biopsy	None	The importance of serum miR‐122 and FIB‐4 index as risk factors for mortality in Japanese patients with histopathologically confirmed NAFLD is shown.	6
Ezaz et al, 2020^43^	50.6, n = 182, USA	Cross‐sectional	NAFLD and NASH	RT‐qPCR and liver biopsy	None	miR‐34a, miR‐122, miR‐192, and miR‐200a demonstrate strong associations with NAFLD severity by histology, but differential associations with pathogenic factors.	6
LncRNAs and fatty liver disease measured in both blood samples and liver tissue							
Sookoian et al, 2017^45^	50, n = 486 Argentina	Case‐control	NAFLD and NASH	Next‐generation sequencing and liver biopsy	Age, sex and BMI	genetic variation in lncRNAs may contribute to the disease severity, rs2829145 was significantly associated with NAFLD as well as the disease severity.	8

Abbreviations: ALT, Alanine transaminase; AST, Aspartate transaminase; AUC, area under the curve; CHB, chronic Hepatitis B; CK18, Keratin 18; eGFR, estimated glomerular filtration rate; FL, fatty liver; GGT, gamma‐glutamyl transferase; HbA1c, Haemoglobin A1c; HDL, high‐density lipoprotein; ISH, in situ hybridization; LDL‐c, low‐density lipoprotein cholesterol; lncRNAs, long noncoding RNAs; miRNAs, microRNAs; MRI, magnetic resonance imaging; NAFLD, nonalcoholic fatty liver disease; NASH, nonalcoholic steatohepatitis; qRT‐PCR, quantitative real‐time polymerase chain reaction; RT‐qPCR, real‐time quantitative polymerase chain reaction; SBP, systolic blood pressure; T2D, type 2 diabetes; TG, triglyceride; VLDL‐C: very low‐density lipoprotein cholesterol.

^a^ Quality assessment based on the Newcastle‐Ottawa Scale. Range 0‐9, higher score is higher quality.

### 3.2 DNA methylation

#### 3.2.1 Global DNA methylation studies

Two studies^16^, ^17^ examined the association between global DNA methylation and FLD (Table 1). One study^16^ conducted among South American participants (normal livers [n = 18], simple steatosis [n = 23] and NASH [n = 22]) used liver tissue samples to evaluate the status of cytosine methylation at the 5mC of liver mitochondrial DNA (mtDNA) in selected regions of the mtDNA genome. This study found that mitochondrial encoded NADH dehydrogenase 6 (MT‐ND6) methylation was higher in the liver of NASH than participants with simple steatosis (*P* < .04). Moreover, the methylation level of MT‐ND6 was significantly associated with NAFLD activity score which was used to evaluate the spectrum of NAFLD (*P* < .02). The other study^17^ conducted among 95 European participants reported that global liver methylation based on genome‐wide methylation arrays was not associated with NAFLD nor NASH. However, when assessed by long‐interspersed nuclear element (LINE‐1) methylation levels, liver global DNA methylation was associated with hypomethylation among participants with NASH as compared to those with NAFLD or healthy controls.

#### 3.2.2 Candidate‐based DNA methylation studies

Five studies^18^, ^19^, ^20^, ^21^, ^22^ examined the relation of FLD with methylation sites in or near candidate genes (Table 1). Overall, these studies reported that methylated CpG sites annotated to *PPARGC1A*, *TFAM*, *FGFR2*, *MAT1A*, *CASP1*, *PARVB*, *PNPLA3*, *PPARα*, *TGFβ1*, *Collagen 1A1, PDGFα, PAPLN*, *LBH, DPYSL3*, *JAG1*, *NPC1L1*, *STARD* and *GRHL* are associated with FLD. An overview of these genes, the association with FLD and function is provided in Table S4. Of these, one study^19^ was performed only in peripheral blood samples, three studies^20^, ^21^, ^22^ only used liver tissue samples and one study^18^ used both blood and liver tissue samples. Two studies used the bisulphite pyrosequencing method,^20^, ^22^ two other studies used methylation‐specific polymerase chain reaction,^18^, ^19^ and one study used targeted‐bisulphite sequencing to quantify DNA methylation.^21^ The majority (n = 4) of these studies reported adjustment or control for confounders.

The five studies performed a candidate gene approach and there was no any overlap between them. These studies found that NAFLD was associated with hypomethylation at *FGFR2*, *MAT1A*, *CASP1* ^20^ and *PARVB* genes^21^ and hypermethylation at *PNPLA3*,^21^ *PPARα*, *TGFβ1*, *Collagen 1A1* and *PDGFα* genes.^22^ One additional study^18^ found that *PPARGC1A* methylation status was significantly associated with NAFLD, and 47.9% of alleles were methylated in participants with NAFLD vs 30.6% in healthy controls (*P* < .01). In addition, no association was found between the methylation status of *GSTT1, GSTP1* ^19^ and *SAMM50* genes^21^ and NAFLD.

#### 3.2.3 Epigenome‐wide DNA methylation studies

Four studies examined the association between DNA methylation and FLD using an EWAS approach. All these studies used illumina Human Methylation 450 (450K) Beadchip to quantify DNA methylation. Two studies^14^, ^23^ used whole blood samples, and the other two studies^24^, ^25^ were performed in liver tissue. Three studies^14^, ^23^, ^24^ adjusted for potential confounders and only one study^25^ did not adjust for any confounders. Two of these studies conducted very recently^14^, ^23^ reported an association between cg06690548 (*SLC7A11*) and FLD.

One study, using whole blood samples^14^ included 4525 individuals from four population‐based cohort studies and the analyses were adjusted for age, sex, smoking status, physical activity levels, alcohol intake and BMI. DNA methylation was assessed at over 400 000 CpGs in whole blood or CD14 + monocytes using a commercial array. They identified 22 CpGs associated with hepatic fat in European ancestry and further performed Mendelian randomization analyses which supported the association of hypomethylation of cg08309687 (*LINC00649*) with NAFLD (*P* = 2.5 × 10^−4^). Another one study^23^ showed that peripheral blood‐derived DNA hypermethylation at one CpG site (cg06690548) located in an intron of *SLC7A11* may be associated with reduced risk of hepatic steatosis. Another study^25^ was conducted among 60 participants [(mild NAFLD (n = 39), advanced NAFLD (N = 21)], found that a total of 1777 genes were differentially expressed between mild and advanced NAFLD cases (q‐value < 0.05) clustered into four modules. One of the modules formed a scale‐free network containing four hub genes (*PAPLN*, *LBH*, *DPYSL3* and *JAG1*) that were overexpressed in advanced NAFLD. Another module formed a random network and was enriched for genes that accumulate in the mitochondria and the other two modules did not form unambiguous network. Lastly, a study^24^ conducted among 178 individuals in Europe, also found that NAFLD is associated with methylation shifts relevant for the expression of three genes (*NPC1L1*, *STARD* and *GRHL*) involved in lipoprotein particle composition.

### 3.3 Noncoding RNAs

MiRNAs are deregulated in NAFLD and have been proposed as useful biomarkers for the diagnosis and stratification of disease severity of NAFLD and NASH.^5^ We found 24 studies^2^, ^6^, ^7^, ^8^, ^9^, ^44^ that investigated the association of miRNAs with FLD (Table 2). Of these, 13 studies used blood samples,^6^, ^37^ three studies used liver tissue^2^, ^38^, ^39^ and eight studies used both blood and liver tissue samples.^7^, ^8^, ^9^, ^40^, ^41^, ^42^, ^43^, ^44^ In addition, the studies included population with Asian (n = 9), European (n = 7), North American (n = 6) and South American (n = 2) ancestries with mean age of 46.9 years old. Overall, data were available on 5288 participants, from which there were 1359 NAFLD cases, 819 NASH cases and 174 simple steatosis cases.

Overall, these studies reported 34 miRNAs associated with FLD (Table 3). Among these, miR‐122 (n = 14), miR‐34a (n = 8), miR‐192 (n = 4), miR‐21 (n = 2) and miR‐99a (n = 2) were associated with FLD in two or more independent studies. Other studies reported that the following miRNAs including miR‐451, miR‐103, miR‐855‐5p, miR‐331‐3p, miR‐30c, miR‐29b, miR‐125b,miR‐16, miR‐7‐1, miR‐29c‐3p, miR‐33b*, miR‐132, miR‐150, miR‐433, miR‐28‐3p, miR‐511, miR‐517a, miR‐671, miR‐181d, miR‐197, miR‐146b, miR‐10b, miR‐29a, miR‐19a, miR‐19b, miR‐375, miR‐20a, miR‐27a and miR‐200a were also linked to FLD.

**TABLE 3:** Deregulated miRNA in liver tissue and blood circulation of participants with fatty liver disease

Liver tissue miRNAs				Circulating miRNAs			
miRNA	Expression	Phenotype	Studies and years	miRNA	Levels	Phenotype	Studies and years
miR‐122	↓	NAFLD and NASH	Braza‐Boïls et al, 2016	miR‐122	↑	NAFLD	Yamada et al, 2013
	↓	NAFLD	Auguet et al, 2016		↑	NAFLD and NASH	Becker et al, 2015
	↔	NAFLD	Salvoza et al, 2016		↑	NAFLD and FLD	Raitoharju et al, 2016
miR‐34a	↑	NAFLD and NASH	Braza‐Boïls et al, 2016		↑	NAFLD	Abdel‐Hamed et al, 2017
	↔	NAFLD	Salvoza et al, 2016		↑	NAFLD	Brandt et al, 2018
miR‐125b	↓	NAFLD and NASH	Sharma et al, 2013		↑	NAFLD	Hendy et al, 2019
miR‐16‐2	↑	NAFLD and NASH	Sharma et al, 2013		↑	NASH and SS	Pirola et al, 2015
miR‐7‐1	↑	NAFLD and NASH	Sharma et al, 2013		↑	NAFLD and NASH	Liu et al, 2016
miR‐33b*	↑	NAFLD and NASH	Auguet et al, 2016		↑	NAFLD	Salvoza et al, 2016
miR‐29c‐3p	↓	NAFLD and NASH	Braza‐Boïls et al, 2016		↔	NAFLD	Delik et al, 2020
miR‐132	↓	NAFLD and NASH	Estep et al, 2010		↑	NAFLD	Akuta et al, 2020
miR‐150	↓	NAFLD and NASH	Estep et al, 2010		↑	NAFLD and NASH	Ezaz et al, 2020
miR‐433	↓	NAFLD and NASH	Estep et al, 2010	miR‐34a	↑	NAFLD	Yamada et al, 2013
miR‐28‐3p	↓	NAFLD and NASH	Estep et al, 2010		↔	NAFLD and NASH	Celikbilek et al, 2014
miR‐511	↓	NAFLD and NASH	Estep et al, 2010		↑	NAFLD	Muangpaisarn et al, 2017
miR‐517a	↓	NAFLD and NASH	Estep et al, 2010		↑	NAFLD and NASH	Liu et al, 2016
miR‐671	↓	NAFLD and NASH	Estep et al, 2010		↑	NAFLD	Salvoza et al, 2016
					↑	NAFLD	Hendy et al, 2019
					↑	NAFLD and NASH	Ezaz et al, 2020
				miR‐21	↑	NAFLD	Yamada et al, 2013
					↑	NAFLD and NASH	Becker et al, 2015
				miR‐451	↑	NAFLD	Yamada et al, 2013
				miR‐192	↑	NAFLD and NASH	Becker et al, 2015
					↑	NASH and SS	Pirola et al, 2015
					↑	NAFLD and NASH	Ezaz et al, 2020
					↓	NAFLD	Hu et al, 2020
				miR‐103	↑	NAFLD	Xu et al, 2015
				miR‐331‐3p	↓	NAFLD	Zarrinpar et al, 2016
				miR‐30c	↓	NAFLD	Zarrinpar et al, 2016
				miR‐885‐5p	↑	NAFLD and FLD	Raitoharju et al, 2016
				miR‐29b	↑	NAFLD	He et al, 2019
				miR‐20a	↓	NAFLD	Ando et al, 2019
				miR‐27a	↓	NAFLD	Ando et al, 2019
				miR‐181d	↓	NAFLD and NASH	Celikbilek et al, 2014
				miR‐99a	↓	NAFLD and NASH	Celikbilek et al, 2014
					↓	NAFLD	Hendy et al, 2019
				miR‐197	↓	NAFLD and NASH	Celikbilek et al, 2014
				miR‐146b	↓	NAFLD and NASH	Celikbilek et al, 2014
				miR‐10b	↓	NAFLD and NASH	Celikbilek et al, 2014
				miR‐29a	↓	NAFLD and NASH	Celikbilek et al, 2014
				miR‐200a	↑	NAFLD and NASH	Ezaz et al, 2020
				miR‐19a	↑	NASH and SS	Pirola et al, 2015
				miR‐19b	↑	NASH and SS	Pirola et al, 2015
				miR‐375	↑	NASH and SS	Pirola et al, 2015

Abbreviations: FLD, fatty liver disease; miRNAs, microRNAs; NAFLD, nonalcoholic fatty liver disease; NASH, nonalcoholic steatohepatitis; SS, simple steatosis.

MiR‐122 is abundant in liver and its function has been extensively studied.^44^ Most of the studies (n = 14) included in this review reported that miR‐122 was associated with FLD and could be used as biomarker for FLD. Among these, 12 studies^6^, ^35^, ^36^, ^41^ were measured in blood samples. Among eleven of these studies indicating that miR‐122 was upregulated in participants with FLD, only one study^35^ found no significant association between circulating miR‐122 and participants with NAFLD. Three studies^2^, ^7^, ^39^ were performed using liver tissue, from which two of these studies^2^, ^39^ found that miR‐122 was downregulated in participants with fatty liver disease compared to healthy controls, and one study^7^ found that the level of miR‐122 did not significantly differ between participants with NAFLD and healthy controls. However, the receiver operating characteristic (ROC) curve analysis revealed that miR‐122 could be a potential marker for discriminating NAFLD patients from healthy controls with an area under the curve (AUC) value of 0.858.^7^ Moreover, a two‐stage study,^40^ investigating a large panel of circulating miRNAs at different phases of NAFLD, showed that circulating miR‐122 was increased by 7.2‐fold in participants with NASH vs healthy controls and 3.1‐fold in participants with NASH vs simple steatosis.

MiR‐34a is weakly expressed in hepatocytes, but 7 studies reported that circulating miR‐34a in blood was significantly upregulated in participants with FLD. One study^9^ conducted among 40 Turkish participants, showed that circulating miR‐34a was not significantly associated with NAFLD or NASH. Another study^7^ conducted among 64 American participants, found that the level of miR‐34a did not significantly differ between participants with NAFLD and healthy controls, but ROC curve analysis revealed that miR‐34a could be a potential marker for discriminating NAFLD patients from healthy controls with an AUC value of 0.781.

MiR‐192 was reported by three studies^26^, ^41^, ^44^ showing that circulating miR‐192 is upregulated in participants with NAFLD, NASH or simples steatosis than healthy controls, only one study^36^ suggested that serum expression of miR‐192‐5p in patients with acute pancreatitis and NAFLD is significantly down‐regulated compared to acute pancreatitis patients without NAFLD and healthy controls.

Of note, two studies^6^, ^27^ have also reported miRNAs to be used as therapeutic targets for the treatment of fatty liver disease without any general overlap between them. One study^6^ reported that miR‐34a plays a role of physiological significance in the biology of NAFLD and may present a therapeutic target for NAFLD. The other study^27^ reported that miR‐103 may be a link between insulin resistance and NAFLD and could be used as a therapeutic target for the treatment of NAFLD.

Additionally, lncRNAs that cover a significant portion of noncoding transcriptome in mammalian genomes, regulate critical aspects of the genome biology.^10^ However, the role of genomic regions encoding lncRNAs in the risk of FLD remains largely unexplored. We identified only one study^45^ that conducted among 486 individuals and hypothesized that variants in lncRNAs could influence the susceptibility to NAFLD. These findings suggested that genetic variation at rs2829145 in lnc‐JAM2‐6 may contribute to the disease severity.^45^

### 3.4 Histone modification

We did not identify any study investigating the association of histone modification with fatty liver disease on humans. Future studies should elucidate whether histone modifications play any possible role in the physiopathology of fatty liver disease as well as in disease prognosis and treatment.

## 4 DISCUSSION

The present study aimed to provide a comprehensive review of the currently available evidence on the role of epigenetic modifications in FLD. Of the 36 included publications, the majority of the studies focused on association of miRNAs with NAFLD and some had a well‐conducted cohort study design, with different tissues and analytical approaches. These results provide substantially support the existence of association between epigenetic alterations and risk of FLD. Yet, due to the small sample size, these findings should be interpreted with caution.

Overall the findings of this review suggest no consistent associations with FLD in the studies of the global DNA methylation. Global DNA methylation provides an assessment of DNA methylation levels in the evaluated tissue sample by quantifying the methylcytosine (5‐mC) present in the genome.^15^ One study^16^ identified *MT‐ND6* methylation was higher and the other study^17^ identified *LINE‐1* was hypomethylated in the livers of participants with NASH compared to participants with simple steatosis or normal livers. Liver *MT‐ND6* mRNA expression was significantly decreased in NASH patients and the status of liver *MT‐ND6* methylation in NASH group was inversely correlated with the level of regular physical activity. Hepatic methylation and transcriptional activity of the *MT‐ND6* are associated with the histological severity of NAFLD. This suggests that epigenetic changes of mtDNA could be potentially reversed by interventional programs, and physical activity could modulate the methylation status of *MT‐ND6*. Moreover, *LINE‐1* may induce genetic variation and polymorphism through the recombination and rearrangement as well as through endogenous mutagenesis, thereby influencing the expression status of genes.

Associations of gene‐specific DNA methylation (candidate‐based approach) with FLD were explored in a few studies and without was found between the significant genes differentially methylated on studies that used this approach. Moreover, two EWAS^14^, ^23^ conducted among 5975 participants which reported an association between cg06690548 (*SLC7A11*) and FLD. Compared to candidate‐based approach examine DNA methylation at specific CpG sites or regions, EWAS are typically hypothesis‐free and screen up to hundreds of thousands of locus across the genome to identify CpGs or regions associated with FLD. In contrast, candidate gene DNA methylation analyses target loci in a limited number of specific genes, based on a priori hypotheses in small sample sizes. The majority of candidate gene studies did not adjust for confounders. There are also some limitations need to be considered on EWAS. Some studies using an EWAS approach in whole blood samples for quantification of DNA methylation might have missed CpG sites that are expressed only in other tissues such as liver.

In this systematic review, most of the epigenetic studies (n = 24) focused on miRNAs and fatty liver disease, but only 13 studies^2^, ^42^ were adjusted or matched for the relevant confounders. In line with a previous meta‐analysis,^5^ our findings suggest an inconsistent or even inverse correlation of the direction of miRNA expression between blood or serum samples and liver tissue samples. For instance, serum miR‐122 was always upregulated in participants with NAFLD or NASH vs healthy controls,^6^, ^26^, ^31^, ^32^, ^41^ but it was unchanged in liver tissue^7^ or even downregulated in liver tissue.^2^, ^39^ Serum miR‐34a level was upregulated in participants with NAFLD or NASH vs healthy controls,^6^, ^7^, ^8^, ^42^ but it was unchanged in liver tissue.^7^

Additionally, a small set of studies included in this review suggested that miRNAs could be used as potential therapeutic targets of FLD. miRNA‐based therapeutics include miRNA mimetics, anti‐miRNA oligonucleotides and exosomes loaded with miRNAs.^46^ Although no miRNAs are in clinical trials for FLD, a few are already in trials for viral hepatitis which may lead to FLD and liver cancer. For instance, several miRNA‐targeted therapeutics have reached clinical development, including molecules targeted at miR‐122, which reached phase II trials for treating hepatitis C,^47^ and a mimic of the tumour suppressor miRNA miR‐34, which reached phase I clinical trials for solid tumours (eg liver).^48^

The current evidence reveals that several differentially methylated sites, such as cg06690548 annotated to *SLC7A11* gene associated with FLD. Most of the CpG sites were involved in lipid metabolism through inducing the expression of lipid‐related genes, but one EWAS showed the FLD associated CpG sites also relation with glucose metabolism.^14^ Moreover, miR‐122, miR‐34a and miR‐192 may play a role in the development of FLD, but the quality of these studies should be considered for interpreting the findings. There are several components that determine the quality of the studies, such as design, sample size, use of tissue, confounder adjustment and replication.

Epigenetic modifications are relatively stable alterations that can explaining the effect of environmental factors on phenotype, and part of the missing heritability of common diseases such as fatty liver disease, which is not accounted for by common genetic variants.^4^ The study of epigenetic markers is emerging as one of the most promising molecular strategies for diagnosis and treatment of FLD. Peripheral blood is easy to access and reflects multiple metabolic and inflammatory pathways. Therefore, methylation profiling in peripheral blood and noncoding RNAs to identify FLD is of great interest since several epigenetic‐based drugs and diagnostic biomarkers have entered clinical development. For example, clustered regularly interspaced short palindromic repeats (CRISPR), to modify the epigenetic control of gene expression for therapeutic purpose has been vastly explored in the last decade.^47^ However, physiological changes as a consequence of increased physical activity and diet changes may also impact DNA methylation activity. For instance, increasing exercise and a low‐carbohydrate diet may improve peripheral insulin resistance, therefore it may reduce the excess delivery of free fatty acids, glucose for free fatty acid synthesis to the liver, and may also impact patterns of DNA methylation.^49^

Collectively, current evidence suggests an association between epigenetic modifications and FLD. Yet, the available research is limited and hampered by small samples, suboptimal designs and heterogeneity in approaches, analyses and tissues. Therefore, more research is needed in the future in order to draw stronger conclusions on the likely complex association between epigenetics and FLD and also decipher molecular pathway by which the epigenetic markers may regulate FLD. Specifically, more studies should examine global, candidate gene DNA methylation and histone modifications in large samples and these findings should be replicated in other populations. Furthermore, longitudinal studies and genetic sensitive designs are needed to examine temporal relation of epigenetics and their causal association with FLD.

## 5 CONCLUSIONS

In conclusion, promising results have been reported in the field of FLD and epigenetics, but still more basic and translational research is needed to understand the causal role of epigenetic modifications in FLD. These findings could pave the way for future studies and ultimately lead to targeted screening of high‐risk individuals in clinical practice. This could be beneficial for both patient stratification for clinical trials, as well as prognostication and treatment when new therapies become available. Nonetheless, these findings should be considered cautiously given the sample sizes of the studies and statistical power, use of different target tissues, precluding solid causal inferences, lack of confounders adjustment and, replication in independent cohorts.

## CONFLICT OF INTEREST

Authors have nothing to disclose.

## AUTHOR CONTRIBUTIONS

The contributions of the authors are as follows: M. Ghanbari, M. Amiri and J. Nano contributed to conceive and design the study, W. M. Bramer contributed to data search strategy, X. Zhang, E. Asllanaj and M. Amiri screened titles/ abstracts. X. Zhang and E. Asllanaj obtained the full‐text, determined the eligibility of articles, data extraction, and assessed the quality of the included studies. X. Zhang and E. Asllanaj participated in data synthesis/ analysis and interpretation of the data. X. Zhang, E. Asllanaj, E. Portilla‐Fernandez and M. Ghanbari drafted the final manuscript. All authors contributed to the critical revision of the manuscript and approved the final version.

## Supporting information

Table S1

Table S2

Table S3

Table S4
