# Supplementary material for: Deciphering the role of epigenetic modifications in fatty liver disease: A systematic review
Source: Eur J Clin Invest. 2021 Jan 4;51(5):e13479. doi: 10.1111/eci.13479 (PMC8243926; doi:10.1111/eci.13479)
Supplement: Supplementary file 2 — Table S2 [file ECI-51-e13479-s003.docx]

**Supplementary Table 2. Search strategy used in the current systematic review**

| **embase.com** |
| --- |
| (epigenetics/exp OR 'DNA methylation'/exp OR 'histone modification'/exp OR 's adenosylmethionine'/exp OR 'CpG island'/exp OR 'microRNA'/exp OR (((histone* OR dna OR 'long interspersed') NEAR/3 (acetylat* OR demethylat* OR methylat* OR phosphorylat* OR ubiquitinat* OR modif*)) OR 's adenosylmethionine' OR cpg OR epigenetic* OR epigenomic* OR microRNA* OR micro-RNA* OR mirna* OR miR OR miRs):ab,ti,kw) AND ('fatty liver'/exp OR 'liver fibrosis'/exp OR 'liver cirrhosis'/exp OR 'alcohol liver disease'/exp OR (('liver disease'/de OR 'liver injury'/de) AND alcoholism/exp) OR (((fat* OR steato* OR fibros* OR cirrho*) NEAR/3 (liver* OR hepat*)) OR steatohepatit* OR hepatosteatos* OR nafl OR nafld OR nash OR ((alcohol* OR nonalcohol*) NEAR/3 (liver OR hepat*) NEAR/3 (injur* OR damage* OR disease*))):ab,ti,kw) NOT ([Conference Abstract]/lim OR [Note]/lim OR [Editorial]/lim) AND [english]/lim NOT ([animals]/lim NOT [humans]/lim) |
| **Medline Ovid** |
| (Epigenomics/ OR DNA methylation/ OR S-Adenosylmethionine/ OR CpG Islands/ OR exp microRNAs/ OR (((histone* OR dna OR long interspersed) ADJ3 (acetylat* OR demethylat* OR methylat* OR phosphorylat* OR ubiquitinat* OR modif*)) OR s adenosylmethionine OR cpg OR epigenetic* OR epigenomic* OR microRNA* OR micro-RNA* OR mirna* OR miR OR miRs).ab,ti,kf.) AND (exp fatty liver/ OR exp Liver Cirrhosis/ OR Liver Diseases, Alcoholic/ OR ((liver diseases/) AND Alcoholism/) OR (((fat* OR steato* OR fibros* OR cirrho*) ADJ3 (liver* OR hepat*)) OR steatohepatit* OR hepatosteatos* OR nafl OR nafld OR nash OR ((alcohol* OR nonalcohol*) ADJ3 (liver OR hepat*) ADJ3 (injur* OR damage* OR disease*))).ab,ti,kf.) NOT (news OR comment OR editorial OR congresses OR abstracts).pt. AND english.la. NOT (exp animals/ NOT humans/) |
| **Cochrane CENTRAL** |
| ((((histone* OR dna OR 'long interspersed') NEAR/3 (acetylat* OR demethylat* OR methylat* OR phosphorylat* OR ubiquitinat* OR modif*)) OR 's adenosylmethionine' OR cpg OR epigenetic* OR epigenomic* OR microRNA* OR micro-RNA* OR mirna* OR miR OR miRs):ab,ti,kw) AND ((((fat* OR steato* OR fibros* OR cirrho*) NEAR/3 (liver* OR hepat*)) OR steatohepatit* OR hepatosteatos* OR nafl OR nafld OR nash OR ((alcohol* OR nonalcohol*) NEAR/3 (liver OR hepat*) NEAR/3 (injur* OR damage* OR disease*))):ab,ti,kw) |
| **Web of science** |
| TS=(((((histone* OR dna OR "long interspersed") NEAR/2 (acetylat* OR demethylat* OR methylat* OR phosphorylat* OR ubiquitinat* OR modif*)) OR "s adenosylmethionine" OR cpg OR epigenetic* OR epigenomic* OR microRNA* OR micro-RNA* OR mirna* OR miR OR miRs)) AND ((((fat* OR steato* OR fibros* OR cirrho*) NEAR/2 (liver* OR hepat*)) OR steatohepatit* OR hepatosteatos* OR nafl OR nafld OR nash OR ((alcohol* OR nonalcohol*) NEAR/2 (liver OR hepat*) NEAR/2 (injur* OR damage* OR disease*)))) NOT ((animal* OR rat OR rats OR mouse OR mice OR murine OR dog OR dogs OR canine OR cat OR cats OR feline OR rabbit OR cow OR cows OR bovine OR rodent* OR sheep OR ovine OR pig OR swine OR porcine OR veterinar* OR chick* OR zebrafish* OR baboon* OR nonhuman* OR primate* OR cattle* OR goose OR geese OR duck OR macaque* OR avian* OR bird*) NOT (human* OR patient*))) AND DT=(article) AND LA=(english) |
| **Google scholar** |
| "histone\|dna methylation\|modification"\|epigenetics\|microRNA\|mirna "fatty liver"\|steatohepatitis\|hepatosteatosis\|nafl\|nafld\|nash\|"alcoholic\|nonalcoholic liver\|hepatic injury\|damage\|disease" |
