# Supplementary material for: Deciphering the role of epigenetic modifications in fatty liver disease: A systematic review
Source: Eur J Clin Invest. 2021 Jan 4;51(5):e13479. doi: 10.1111/eci.13479 (PMC8243926; doi:10.1111/eci.13479)
Supplement: Supplementary file 3 — Table S3 [file ECI-51-e13479-s002.docx]

**Supplementary Table 3. Selection Criteria used in the current systematic review**

| **Inclusion criteria** |
| --- |
| 1. Include cross-sectional, prospective, cohort, case-cohort and nested case-control studies, case-control.  2. Include studies which described:  • An association between epigenetic marks (Global, site specific or Genome-wide methylation of DNA) and fatty liver disease ( NAFLD, hepatic steatosis, hepatic fat, fatty liver, simple steatosis and NASH)  • An association between histone Modifications (methylation, phosphorylation, acetylation, ubiquitylation and sumoylation) and fatty liver disease.  • An association between non-coding RNAs (miRNAs and lncRNAs) and fatty liver disease.  3. Include studies conducted in humans.  4. No language or date restriction. |
| **Exclusion criteria** |
| 1. Exclude abstracts, cost effectiveness studies, letters to the editor, conference proceedings, systematic reviews or meta-analyses.  2. Exclude studies conducted in animals. |

Abbreviations : NAFLD, non-alcoholic fatty liver disease; NASH, non-alcoholic steatohepatitis; miRNAs, microRNA; lncRNAs, long non-coding RNAs.
