# Supplementary material for: Deciphering the role of epigenetic modifications in fatty liver disease: A systematic review
Source: Eur J Clin Invest. 2021 Jan 4;51(5):e13479. doi: 10.1111/eci.13479 (PMC8243926; doi:10.1111/eci.13479)
Supplement: Supplementary file 4 — Table S4 [file ECI-51-e13479-s004.docx]

**Supplementary Table 4. Functional annotation of genes differentially methylated with fatty liver disease**

| **Gene** | **Gene name** | **Gene function** |
| --- | --- | --- |
| **PPARGC1A** | peroxisome proliferator–activated receptor ɣ coactivator 1α | plays an essential role in metabolic reprogramming in response to dietary availability through coordination of the expression of a wide array of genes involved in glucose and fatty acid metabolism |
| **TFAM** | Mitochondrial transcription factor A | a key activator of mitochondrial transcription, binds to the mitochondrial light strand promoter and functions in mitochondrial transcription regulation |
| **FGFR2** | Fibroblast growth factor receptor 2 | acts as cell-surface receptor for fibroblast growth factors and plays an essential role in the regulation of cell proliferation, differentiation, migration and apoptosis, and in the regulation of embryonic development |
| **MAT1A** | Methionine adenosyl methyltransferase 1A | a target of promoter methylation in hepatocarcinogenesis |
| **CASP1** | Caspase 1 | releasing the mature cytokine which is involved in a variety of inflammatory processes. Important for defense against pathogens and cleaves and activates sterol regulatory element binding proteins |
| **PARVB** | Beta-parvin | involved in the reorganization of the actin cytoskeleton and formation of lamellipodia. Plays a role in cell adhesion, cell spreading, establishment or maintenance of cell polarity, and cell migration. |
| **PNPLA3** | Patatin-like phospholipase domain containing 3 | regulate the development of adipocytes and the production and breakdown of fats (lipogenesis and lipolysis) in hepatocytes and adipocytes. |
| **PPARα** | Peroxisome proliferator-activated receptor alpha | involved in DNA-binding transcription activator activity, lipid binding |
| **TGFβ1** | Transforming growth factor beta 1 | involved in growth factor activity |
| **Collagen 1A1** | alpha 1 type I collagen | involved in extracellular matrix structural constituent |
| **PDGFα** | Platelet-derived growth factor-alpha | involved in growth factor activity |
| **PAPLN** | Papilin, proteoglycan-like sulfated glycoprotein | involved in peptidase activity and serine-type endopeptidase inhibitor activity, stops, prevents or reduces the activity of serine-type endopeptidases |
| **LBH** | Limb bud and heart development | involved in mammary gland epithelial cell differentiation, negative regulation of intracellular estrogen receptor signaling pathway, stem cell differentiation, transcription |
| **DPYSL3** | Dihydropyrimidinase-like 3 | hydrolase activity and phosphoprotein binding |
| **JAG1** | Jagged-1 | helps to determine cellular fate and is active during many developmental stages |
| **NPC1L1** | Niemann-Pick C1-Like 1 | plays a major role in cholesterol homeostasis. critical for the uptake of cholesterol across the plasma membrane of the intestinal enterocyte. the direct molecular target of ezetimibe |
| **STARD** | StAR-related lipid transfer protein | microtubule-dependent motor protein required for spindle pole assembly during mitosis |
| **GRHL** | Grainyhead Like Transcription | essential developmental transcription factors in epithelial cell morphogenesis/differentiation and in tumorigenesis |
